# A Low-Sugar Flavored Beverage Improves Fluid Intake in Children During Exercise in the Heat

**DOI:** 10.3390/nu17152418

**Published:** 2025-07-24

**Authors:** Sajjad Rezaei, Rocio I. Guerrero, Parker Kooima, Isabela E. Kavoura, Sai Tejaswari Gopalakrishnan, Clarissa E. Long, Floris C. Wardenaar, Jason C. Siegler, Colleen X. Muñoz, Stavros A. Kavouras

**Affiliations:** 1 Hydration Science Lab, College of Health Solutions, Arizona State University, Phoenix, AZ 85004, USA; srezaei8@asu.edu (S.R.); celong4@asu.edu (C.E.L.); 2 Athleat Field Lab, College of Health Solutions, Arizona State University, Phoenix, AZ 85004, USA; rguerr16@asu.edu (R.I.G.); floris.wardenaar@asu.edu (F.C.W.); 3 Integrated Human Performance Lab, College of Health Solutions, Arizona State University, Phoenix, AZ 85004, USA; jason.siegler@asu.edu; 4 Hydration Health Center, University of Hartford, West Hartford, CT 06117, USA

**Keywords:** palatability, drinking, body fluid balance, sweating, dehydration

## Abstract

**Objectives**: This study examined the impact of a low-sugar flavored beverage on total fluid intake and hydration biomarkers during intermittent exercise in a hot environment among healthy children. **Methods**: Twenty-one children (11 girls, 8–10 y) completed a randomized, crossover study with two trials. Each trial involved three bouts of 10 min walking, 5 min rest, 10 min walking, and 35 min rest for a total of 3 h in a hot (29.9 ± 0.6 °C) and dry environment (26 ± 7% relative humidity). Walking intensity was 69 ± 7% of age-predicted maximum heart rate. Participants consumed either plain water (W) or a low-sugar flavored beverage (FB). Body weight, fluid intake, urine samples, and perceptual ratings were collected. **Results**: Total ad libitum fluid intake was significantly higher with the FB (946 ± 535 mL) than with W (531 ± 267 mL; *p* < 0.05). This difference was 128% higher for FB compared to W, with 19 out of the 21 children ingesting more fluids in FB versus W. Children rated the FB as more likable across all time points (*p* < 0.05). Net fluid balance was better with FB at 60, 70, 85, 135, and 145 min (*p* < 0.05), though not different at the 3 h mark. Urine volume was higher with FB (727 ± 291 mL) than with W (400 ± 293 mL; *p* < 0.05). Urine osmolality was significantly higher in the W trial at 120 and 180 min (*p* < 0.05). **Conclusions**: A flavored, low-sugar beverage enhanced ad libitum fluid intake and improved hydration markers compared to water during exercise in the heat, supporting its potential as a practical rehydration strategy for children.

## 1. Introduction

A growing body of evidence highlights the widespread prevalence of inadequate hydration among children. According to 2005–2010 data from the National Health and Nutrition Examination Survey (NHANES), over 75% of children aged 4 to 13 years in the United States did not meet the dietary water intake guidelines set by the Institute of Medicine [1]. Additionally, underhydration (urine osmolality > 800 mmol/kg), was reported in more than half of the children who participated in NHANES 2009–2012 [2]. Supporting these findings on a broader scale, Suh and Kavouras (2019) reported that, on average, 60 ± 24% of children worldwide failed to meet established water intake guidelines [3]. Underhydration in children has been associated with reduced cognitive function [4,5], impaired memory [6], and exercise performance [7]. Notably, even short-term fluid intake interventions have been shown to improve hydration status [8], cognitive function [9], and physical performance [7].

Exercise in a hot environment increases body temperature, facilitating heat dissipation primarily through sweat evaporation [10]. In children, without adequate fluid replacement, this results in measurable body water loss (dehydration) as much as adults [11], impairing thermoregulation [12]. Children are particularly vulnerable to dehydration due to physiological differences in water balance and heat regulation. Compared to adults, they have a higher body surface area relative to body weight, resulting in greater insensible water loss through the skin [13,14]. Their renal function, along with voiding volume and frequency, continues to mature through adolescence, further influencing fluid regulation [15]. During exercise in the heat, children typically exhibit lower maximal aerobic capacity, higher adiposity, and reduced sweat output, limiting their ability to lose heat through evaporation [16,17,18,19]. Considering the unique physiological characteristics of children and the high prevalence of inadequate fluid intake among children, it becomes important to focus on strategies that support adequate hydration. When properly hydrated, children can regulate body temperature and tolerate exercise in the heat as effectively as adults [20].

The American Academy of Pediatrics (2011) recommends children aged 9–12 years drink 100 to 250 mL of fluid every 20 min, and that adolescents drink 1 to 1.5 L per hour to replace fluid loss during exercise [20]. However, studies show that when children are offered only plain water and allowed to drink voluntarily, they fail to drink adequate water and become progressively dehydrated [21,22,23,24,25,26,27,28]. The flavor and drink content can influence ad libitum fluid intake in children, as exercise- and heat-induced dehydration resulted in an increase in thirst perception and drink desirability [28]. Compared to plain water, flavored drinks increased fluid intake in both heat-acclimatized and non-acclimatized boys and girls [24,25,26]; however, only one study reported a significant difference between plain and flavored water trials [26]. Further, adding carbohydrates and sodium to flavored water (like sports drinks) led to even greater fluid intake and helped prevent dehydration in these groups [22,24,25,26]. The added sugar sweeteners in carbohydrate-sodium beverages likely improve palatability, and the sodium content may further improve the taste and stimulate the sensation of thirst [29], together promoting voluntary fluid intake.

Despite evidence that carbohydrate and sodium-containing beverages can improve hydration during exercise, the high intake of sugar-sweetened beverages (SSBs) among children remains a significant public health concern [30,31] due to their associations with obesity, dental caries, and emerging evidence of insulin resistance [31]. Moreover, there is little justification for adding sodium to carbohydrate-sodium beverages [32], as children sweat sodium concentration and sweat rate are both lower than those of adults [19], for whom these beverages were originally formulated. On the other hand, the US Food and Drug Administration (FDA)-approved non-nutritive sweeteners (NNSs) may offer a strategy to increase beverage palatability without adding sugar. In this context, flavoring a beverage sweetened with NNSs may offer a practical solution to encourage ad libitum fluid intake and better support hydration during exercise in children.

The aim of the present study was to examine whether a low-sugar flavored beverage could improve ad libitum fluid intake and hydration compared to water during intermittent walking in children aged 8–10 years. To our knowledge, this study involves the youngest cohort investigated to date in rehydration research during exercise in the heat. We hypothesized that the intake of a low-sugar flavored beverage would result in a higher voluntary fluid intake, a smaller net fluid loss, and lower urine concentration compared to plain water during a three-hour intermittent exercise protocol in children.

## 2. Materials and Methods

### 2.1. Study Design

In this randomized and counterbalanced crossover study, 21 children completed two experimental exercise trials, during which they had ad libitum access to either plain water or a flavored beverage. The order of trials was randomized for each participant using Excel to evenly assign trial sequences, with trials separated by a period of at least one and no more than two weeks.

### 2.2. Participants

The study inclusion criteria were as follows: aged 8–10 years, body weight being less than or equal to the 85th percentile for their age group, and willing to exercise in a warm environment. Volunteers were excluded if they had a history of diabetes or renal disease, used attention-deficit/hyperactivity disorder medication, selective serotonin reuptake inhibitor medication, diuretic drugs, or any other medication that could influence thirst or fluid balance. In addition, subjects were excluded if they had a previous diagnosis of heat stroke, had a current or recent illness involving fever, diarrhea, gastrointestinal discomfort, or had physical or mental disabilities preventing them from participating in moderate-intensity treadmill walking.

Participants were recruited through word of mouth, flyers, social media, and online advertisements and received financial compensation for their participation. Written informed consent was obtained from participant parents/guardians, and informed assent was obtained from participants.

Based on previous work from Rivera-Brown et al. (2008), an effect size of at least d = 0.59 was expected for total ad libitum fluid intake between water consumption and a flavored beverage [25]. This corresponds to a minimum required sample size of *n* = 20 (with α = 0.05 and power (1β error probability) of 0.8).

The study was approved by the institutional review board at Arizona State University (STUDY00017618) for human experimentation in accordance with the Helsinki Declaration of 1975 as revised in 1983. This trial was registered before the onset of subject recruitment at https://clinicaltrials.gov/ (accessed on 22 July 2025) as NCT06084702.

### 2.3. Intervention

The study examined the effects of ad libitum intake of a low-sugar, flavored beverage compared to plain water on total fluid intake and hydration biomarkers in children. During each trial, participants underwent a 3 h walk and rest protocol in a warm environment (29.9 ± 0.6 °C, 26 ± 7% relative humidity), consisting of three repeated cycles of 10 min of exercise, five minutes of rest, 10 min of exercise, and 35 min of rest, totaling 60 min of exercise over the 3 h protocol. During the 3 h protocol, subjects had access to one of the following two fluids per trial: (a) plain water (W) or (b) a commercially available low-sugar flavored beverage (FB; Capri Sun Roaring^®^ Water, fruit punch flavor, sweetened with stevia leaf extract). Table 1 presents the beverage composition.

### 2.4. Outcomes

**Figure 1:**
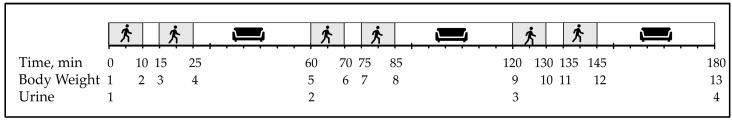
Schematic representation of the protocol. The 3 h trial involved intermittent walking and rest periods in a hot, dry environment (29 °C, 26% RH). Body weight was assessed at 13 time points, and urine samples were collected at four time points to monitor hydration status. Participants had ad libitum access to either plain water or a flavored beverage throughout the protocol.

The study’s primary outcomes were as follows: Total fluid intake (mL) during the 3 h protocol and net fluid balance, calculated from body weight changes over the 3 h walking and rest protocol (Figure 1). Body weight was measured using a calibrated Seca 869 floor digital scale (Seca Ltd., Hamburg, Germany) at multiple time points as shown in Figure 1. Secondary outcomes included urine osmotic excretion (mmol), which was calculated by multiplying urine osmolality (mmol/kg) by urine weight in kg, using the following equation: Urine osmotic excretion = Urine osmolality × Urine weight.

Urine samples were collected at min 0, 60, 120, 180 (Figure 1). Urine volume was estimated by scale, assuming that 1 g equals 1 mL, and aliquots were analyzed for osmolality (A2O Advanced Automated Osmometer, Advanced Instruments Inc., Norwood, MA, USA), specific gravity (PAL-10S Digital Hand-held Urine Specific Gravity Refractometer, Atago, Tokyo, Japan), and urine color [29]. If participants needed to void their bladder between scheduled urine samples, urine was collected in the container scheduled for their next void to contribute to the urine produced between each time point.

Perceptual responses, including likeness, thirst, stomach fullness, taste, and mouth dryness, were assessed at min 0, 25, 60, 85, 120, 145, 180 using a visual analog scale. Each scale was presented to the participants individually in a random order. The visual analog scales consisted of a 175 mm line with an anchor on the left side (0 mm, “not at all”) and a second anchor on the 125 mm mark labeled “extremely”. The “extremely” label (125 mm) was described as “the most they had ever felt in their life”. If participants experienced a perception greater than they had ever felt, they could mark beyond the right bound of 125 or extend the line beyond 175 mm.

### 2.5. Study Protocol

During a screening visit, body weight and height were measured via the ultrasonic measuring station Seca 286 DP (Seca Ltd., Hamburg, Germany). Body fluid compartments for intra- and extracellular fluids were assessed using a multi-frequency bioelectrical impedance spectroscopy (Impedimed SFB7, Carlsbad, CA, USA) unit with dual tab 292-BCE electrodes. The electrodes were placed on the participant’s non-dominant posterior hand and superior foot in a seated position. The characteristics of volunteers in this study are shown in Table 2.

Eligible participants were provided a standardized frozen meal to be consumed as the last meal of the day before each experimental trial. The participants were instructed to consume plenty of fluids to ensure adequate hydration on the morning of the experimental day. After the last meal, participants were instructed not to consume anything other than plain water for eight hours before each trial. Upon arrival, subjects provided an initial urine sample to ensure adequate hydration (urine specific gravity ≤1.020). In cases where a participant presented with a urine specific gravity (USG) above 1.020 on the morning of the trial, they were provided with 500 mL of plain water, and USG was reassessed after 20 min. If the participant still did not meet the hydration criterion, their visit was rescheduled.

After passing the hydration test via USG, the participant was provided with a standardized breakfast consisting of a bagel with one tablespoon of cream cheese and 250 mL of orange juice. Twenty minutes after breakfast, the participants emptied their bladders, and a baseline euhydrated body weight was recorded. Participants began the 3 h walking and resting protocol with ad libitum access to either water or Roarin’ Waters and remained in the hot environment throughout the entire trial (Figure 1). Beverages were served chilled (7 °C) in metal cups with lids and straws to prevent the children from visually identifying the volume of fluids and to avoid the potential influence of beverage packaging on drinking behavior. In each trial, participants completed three 10 min walking sessions alternating with five minutes of sitting, 10 min of walking again, and 35 min of rest for 180 min in the heat (Figure 1). Five minutes were allocated to conducting experimental measures. Exercise was performed on a motorized treadmill at a speed of 5.0 ± 0.6 km/h, corresponding to approximately 69 ± 7% of the age-predicted maximum heart rate. The heart rate during exercise was monitored with a Polar heart rate monitor (Polar, FT1 watch, H1 heart rate sensor, Electro, Oy, Kempele, Finland). All measurements were performed in a warm and dry environment (29.9 ± 0.6 °C, 26 ± 7% relative humidity).

### 2.6. Statistical Analysis

A post hoc sensitivity analysis indicated that with a sample size of 21, the study was powered to detect effect sizes greater than 0.031 (partial eta squared, ηp^2^) with 80% power and α = 0.05, which is comparable with the estimated effect size (ηp^2^ ≈ 0.042; ~181 mL difference in voluntary fluid intake) calculated from data reported by Wilk et al. (2007) when comparing flavored water to plain water in children [26]. Effect sizes were interpreted using thresholds of 0.01, 0.06, and 0.14 to indicate small, medium, and large effects, respectively [33].

All statistical analyses were conducted using JMP 18 (SAS Institute Inc., Cary, NC, USA). Data are presented as mean ± SD, and statistical significance was set at *p* < 0.05. To confirm comparable baseline and environmental conditions across trials, paired *t*-tests were conducted on baseline body weight and USG, as well as mean exercise heart rate, ambient temperature, and relative humidity. Primary and secondary outcomes, including total fluid intake, net fluid balance, urine volume, urine osmolality, osmotic excretion, urine specific gravity, urine color, and perceptual responses, were analyzed using two-way repeated-measures analysis of variance, with beverage condition (plain water vs. flavored beverage), time, and their interaction entered as within-subject fixed effects and participants as a random effect. Mauchly’s test was used to assess sphericity, and the Greenhouse–Geisser correction was applied when the assumption was violated. Following a significant main effect for the analysis of variance, post hoc analyses were conducted using Bonferroni-adjusted multiple comparison tests. Missing data were estimated using regression-based imputation. A comprehensive Table S1 of descriptive statistics (mean, SD) for all variables across time points has been added as a Supplementary Materials.

## 3. Results

There was no significant difference between beverage types in temperature and relative humidity across trials (*p* > 0.05). Exercise intensity was similar across trials, as indicated by non-significant differences in average heart rate percentage (*p* > 0.05). Participants also began each trial with a similar USG and body weight (*p* > 0.05).

### 3.1. Fluid Intake

**Figure 2:**
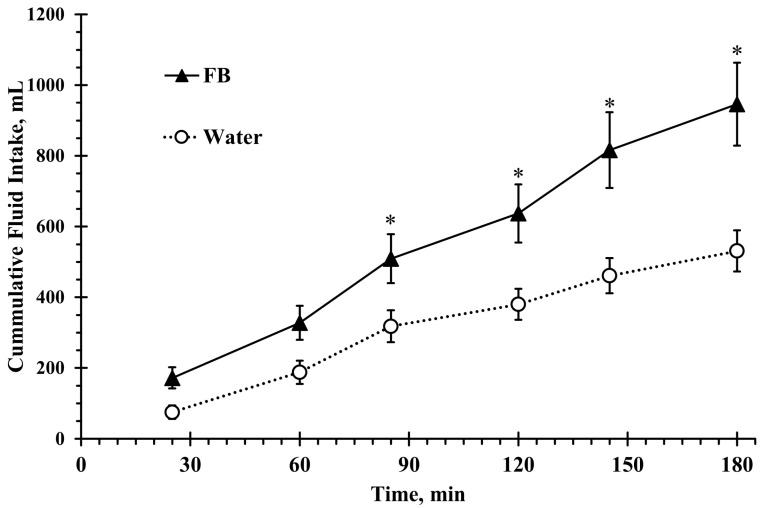
Cumulative fluid intake during the 3 h intermittent exercise in the heat. Participants consumed either a flavored beverage (FB) or plain water ad libitum. Values represent mean ± SE. * denotes statistically significant difference from Water for the same time point (*p* < 0.05).

Total fluid intake over the 3 h rehydration was significantly higher in the flavored beverage trial (946 ± 535 mL) compared to water (531 ± 267 mL), with a mean difference of 415 ± 465 mL (*F*(1, 20) = 14.6, *p* < 0.001, ηp^2^ = 0.42; Figure 2). Eighteen out of the 21 participants ingested more fluids in the favored beverage trial compared to the water one, representing a mean individual-level percent difference of 126% between trials. Fluid intake increased over time (*F*(5, 100) = 10.4, *p* < 0.001, ηp^2^ = 0.78), and post hoc comparison revealed higher fluid intake in FB beginning at 85 min (*p* < 0.05), which became more pronounced at 120 min (*p* < 0.001) and peaked by 180 min (*p* < 0.001; Figure 2).

### 3.2. Net Fluid Balance

**Figure 3:**
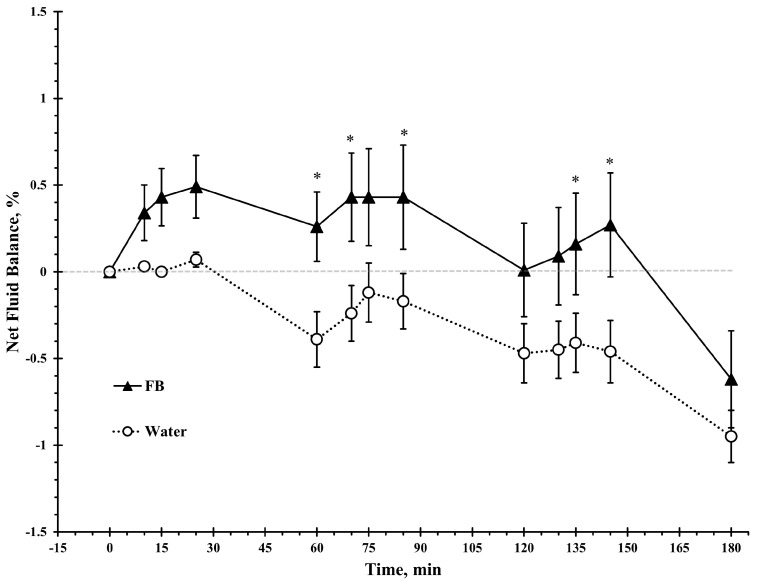
Net fluid balance over time during intermittent exercise in the heat with ad libitum access to either a flavored beverage (FB) or plain water. Values represent mean ± SE. * denotes statistically significant difference from water for the same time point (*p* < 0.05).

A significant main effects of beverage type (*F*(1, 20) = 15.5, *p* < 0.001, ηp^2^ = 0.44), time (*F*(1, 26) = 4.3, *p* < 0.05, ηp^2^ = 0.18), and beverage × time interaction (*F*(3, 61) = 3.9, *p* < 0.05, ηp^2^ = 0.17) was observed for net fluid balance. As shown in Figure 3, there was a significant difference between beverages at 60 min, 70 min, 85 min, 135 min, and 145 min (*p* < 0.05).

### 3.3. Urine Output

**Figure 4:**
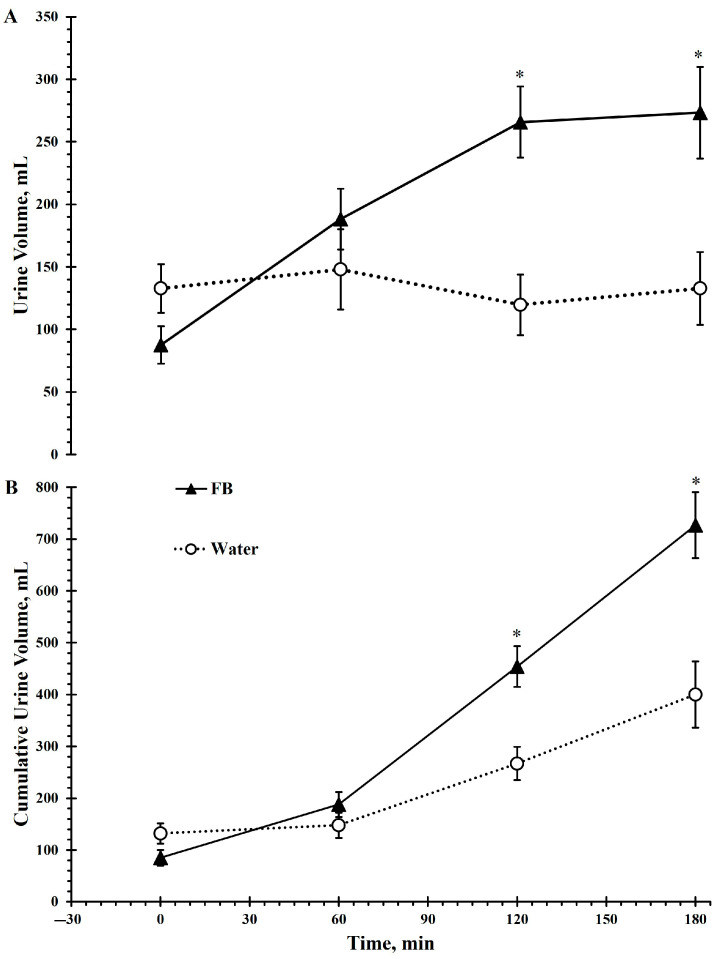
Urine volume per collection (**A**) and cumulative urine volume (**B**) during the 3 h intermittent exercise in the heat with ad libitum access to either a flavored beverage (FB) or plain water. Values represent mean ± SE. * denotes statistically significant difference from Water for the same time point (*p* < 0.001).

Urine volume was significantly influenced by beverage type (*F*(1, 20) = 16, *p* < 0.001, ηp^2^ = 0.44), time (*F*(3, 60) = 4.6, *p* < 0.05, ηp^2^ = 0.19), and beverage × time interaction (*F*(3, 60) = 9.5, *p* < 0.001, ηp^2^ = 0.32). Urine volume increased over time and was consistently higher with FB compared to water at 120 min and 180 min (*p* < 0.001; Figure 4A)

Cumulative urine volume increased across time (*F*(3, 60) = 57.4, *p* < 0.001, ηp^2^ = 0.74), and was greater overall in the flavored beverage (*F*(1, 20) = 18.4, *p* < 0.001, ηp^2^ = 0.48; Figure 4B). A significant beverage × time interaction was also observed (*F*(3, 60) = 24.4, *p* < 0.001, ηp^2^ = 0.74). Post hoc comparison showed that urine accumulation was significantly higher at 120 min (*p* < 0.001) and increased further at 180 min (*p* < 0.001) following the flavored beverage consumption compared to plain water (Figure 4B).

### 3.4. Urine Osmolality

**Figure 5:**
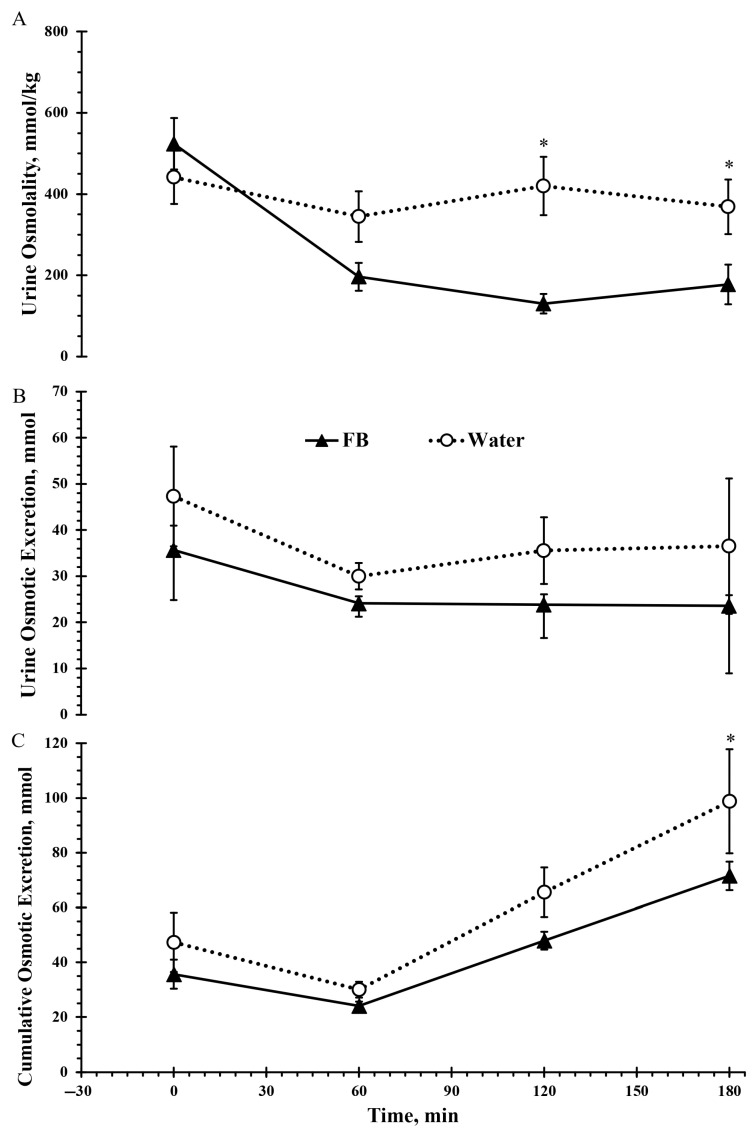
Urine osmolality (**A**), osmotic excretion per collection (**B**), and cumulative osmotic excretion (**C**) during 3 h intermittent exercise in the heat with ad libitum access to either a flavored beverage (FB) or plain water. Values represent mean ± SE. * denotes statistically significant difference from Water for the same time point (*p* < 0.05).

Significant main effects of time (*F*(2, 30) = 5.9, *p* < 0.05, ηp^2^ = 0.26), beverage type (*F*(1, 17) = 11.9, *p* < 0.05, ηp^2^ = 0.41), and a time × beverage interaction (*F*(2, 37) = 5.1, *p* < 0.05, ηp^2^ = 0.23) were detected for urine osmolality. As illustrated in Figure 5A, urine osmolality was significantly higher following W compared to FB at 120 min (*p* < 0.001) and 180 min (*p* = 0.018).

There was no significant difference between beverage types in urine osmotic excretion (*F*(1, 17) = 2.61; *p* = 0.124, ηp^2^ = 0.13; Figure 5B). For cumulative osmotic excretion, time and beverage type were significant (*F*(2, 27) = 18, *p* < 0.001, ηp^2^ = 0.51 and *F*(1, 17) = 7.1, *p* < 0.05; ηp^2^ = 0.21). Figure 5C shows that cumulative osmotic excretion increased as the trial progressed, and a significant difference was observed at 180 min (*p* < 0.05).

### 3.5. Urine Specific Gravity and Color

There were a significant main effects of beverage type (*F*(1, 18) = 10, *p* < 0.05, ηp^2^ = 0.36), time (*F*(2, 31) = 5.6, *p* < 0.05, ηp^2^ = 0.24), and time × beverage interaction (*F*(3, 54) = 4.5, *p* < 0.05, ηp^2^ = 0.21; Table 3) for USG. Specifically, a significantly lower USG in the FB trial was observed at 120 min (*p* < 0.001) and at 180 min (*p* < 0.05).

Urine color in FB became progressively brighter as the trial progressed (Table 3), and a main effect of time (*F*(2, 35) = 5.5, *p* < 0.05, ηp^2^ = 0.24), beverage (*F*(1, 18) = 8.4, *p* = *p* < 0.05, ηp^2^ = 0.32), and time × beverage (*F*(3, 54) = 7.7, *p* < 0.001, ηp^2^ = 0.3). Specifically, significant differences were observed at 120 min (*p* < 0.001), and at 180 min (*p* < 0.05).

### 3.6. Perceptual Ratings

**Figure 6:**
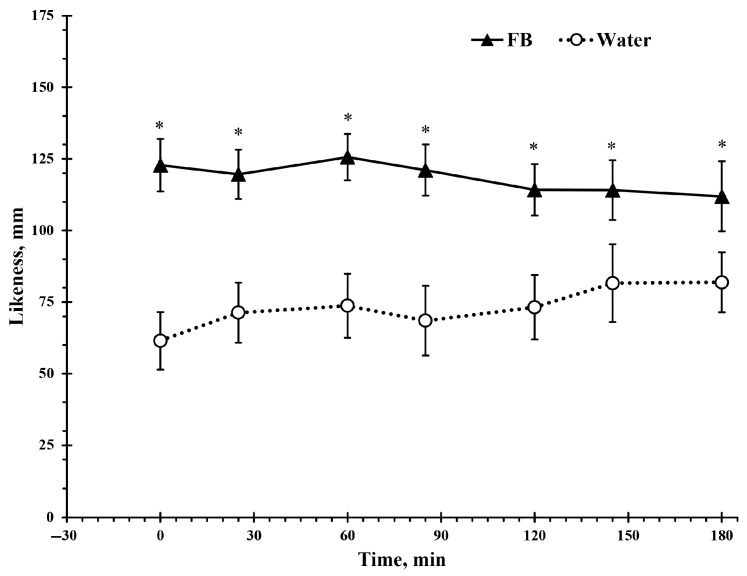
Drink likeness perception was measured using a 175 mm visual analog scale during 3 h intermittent exercise in the heat with ad libitum access to either a flavored beverage (FB) or plain water. Values represent mean ± SE. * denotes statistically significant difference from Water for the same time point (*p* < 0.05).

A significant main effect of beverage type was observed for the taste of the beverage (*F*(1, 20) = 29.8, *p* < 0.001, ηp^2^ = 0.6). Participants rated the flavored beverage as more likable than plain water across all time points, with this preference evident from the beginning to the end of the study (Figure 6).

No significant differences were observed in thirst, mouth dryness, and stomach fullness *p >* 0.05; Table 4).

## 4. Discussion

The results of this study demonstrated that flavoring water with a small amount of sugar and non-caloric sweetener significantly increased ad libitum fluid intake compared to plain water during a 3 h intermittent brisk walking in the heat among children aged 8–10 years. As exercise and heat exposure progressed, fluid consumption increased in both conditions; however, intake in the flavored beverage trial was significantly greater than water from 85 min onward and remained so for the duration of the protocol (Figure 2).

A similar drinking pattern has previously been reported in 9–12-year-old non-acclimatized children [26]. Wilk and Rivera-Brown (2007) observed a 24% increase in fluid intake among girls aged 9–12 years when flavored water was compared to plain water [26]. In contrast, other studies using a comparable protocol found no statistically significant improvement in fluid intake with flavored beverages among heat-acclimatized girls [25], non-acclimatized boys [24], and heat-acclimatized boys and girls [34]. Importantly, the 78% higher fluid intake observed in the present study is not only substantially greater than the increases previously reported but also occurred in 18 out of the 21 children, started earlier, and was sustained throughout the protocol.

The product used in this study contained a very low energy content (121 kJ/L), with 5.7 g/L of sugar, 0.3 g/L of stevia leaf extract, and 3.45 mmol/L of sodium (Table 1). This formulation may partly explain the larger drinking response observed, as the palatability is associated with hedonic value and increased and sustained food and fluid consumption [35]. The addition of non-nutritive sweeteners, such as those used in the current study alongside a negligible amount of sugar, likely enhanced palatability and hedonic appeal [36], thereby stimulating ad libitum fluid intake beyond what is typically achieved through flavoring alone. In contrast, the more modest increases in fluid intake reported in previous studies might reflect the use of plain flavored water without added sweeteners [24], which may have lacked the sensory stimulus to significantly influence voluntary drinking during exercise in the heat.

Moreover, discrepancies in findings may be attributable to differences in environmental conditions, participant characteristics, and study setting. The ambient temperature and relative humidity in previous studies were higher (by approximately 3–5 °C and 10–40%, respectively) than in the present study, and participants in those studies were older than our cohort. While these environmental and age-related differences may have influenced thermoregulatory responses, such as sweating rate and net fluid balance, they are less likely to fully explain the observed differences in voluntary fluid intake. In contrast, a key procedural difference in the present study was the inclusion of an additional 5 min rest period between exercise bouts, which may have provided children with more frequent and accessible opportunities to consume fluids.

Participants in the present study experienced a more favorable net fluid balance throughout the protocol following FB consumption compared to plain water, despite excreting a larger urine volume. This positive net balance was maintained from the beginning of the protocol until the end of the last walking at minute 145 and then declined during the final recovery phase (Figure 3). The observed effect is likely due to greater fluid intake in the FB trial rather than beverage composition, as FB contained only very low amounts of carbohydrate and sodium (Table 2). Previous studies have reported lower urine volume and greater net fluid balance following ad libitum consumption of carbohydrate- and sodium-containing beverages compared to flavored water among children exercising in heat [24,25,26], even when total fluid intake did not differ between trials [26]. Importantly, the present study isolated the influence of beverage palatability, particularly children’s preference for sweet flavors, on fluid intake, independent of carbohydrate as an energy source or sodium as a driver of thirst.

An important observation of this study is that access to fluid in both trials helped prevent underhydration, as indicated by urine osmolality values remaining well below 800 mmol/kg throughout the protocol (Figure 5). Notably, FB resulted in better hydration status at minute 120, which was maintained through the end of the protocol, as reflected by lower USG, urine osmolality, and urine color. These findings support the role of beverage appeal in maximizing fluid intake [37], without increasing sugar content, an important consideration given the association between sugar-sweetened beverage consumption and adverse health outcomes in children [31].

In the United States, beverages are the primary source of total water intake in children, with plain water contributing the most (27.3%) [1]; yet, over 75% of children aged 4–13 years still fail to meet recommended dietary water intake guidelines [1]. Several factors may limit plain water consumption in this age group, including low palatability and widespread availability of more appealing SSBs [38]. The findings of the present study offer a novel strategy to not only promote voluntary water intake but also reduce the consumption of added sugars from sweetened beverages. This study is the first to demonstrate that enhancing water taste by adding a combination of a small amount of carbohydrate and stevia (a natural non-nutritive sweetener approximately 100 times sweeter than sucrose), successfully improves the palatability, resulting in a 78% increase in fluid intake during exercise in the heat. This approach is consistent with the 2015–2020 Dietary Guidelines for Americans, which recommend limiting added sugar intake to less than 10% of total daily energy and selecting beverages with no added sugars [39].

NNSs are widely used in the food supply; however, current evidence on their long-term effects on children remains limited. Inconsistencies across scientific recommendations reflect the lack of conclusive data on their long-term impact in pediatric populations. Nevertheless, stevia has been granted Generally Recognized As Safe status by the FDA and is metabolized similarly in healthy children and adults [40]. Some studies suggest that NNSs may help reduce added sugar intake and support weight management in youth, although more research is warranted to fully understand their long-term role [41]. Importantly, the FB used in the present study contained a small amount of carbohydrate, which reduced the amount of stevia required to enhance beverage palatability. This application focuses on hydration rather than weight control and may represent a practical strategy to increase fluid consumption while minimizing added sugar content in children’s diets.

This study has several limitations. Hydration status was assessed using urine biomarkers only; plasma osmolality or plasma volume changes were not measured. Core temperatures were not evaluated, which limits the interpretation of physiological responses during exercise in the heat. The short-term design prevents conclusions about the long-term effects of repeated use. Additionally, findings may not generalize beyond healthy, prepubertal children.

## 5. Conclusions

In conclusion, a flavored low-sugar beverage improved fluid intake, net fluid balance, and hydration status compared to plain water during intermittent exercise in the heat among children. These findings suggest that palatable, low-sugar drinks may be an effective strategy to promote hydration in active youth without increasing sugar intake within the limitations of the present sample and experimental conditions.

## Figures and Tables

**Table 1:** Experimental Drinks Composition.

Drink	Sodium mmol/L	Potassium mg/L	Carbohydrates g/L	Energy kJ/L	Osmolality mmol/kg H_2_O
FB	3.45	0	5.7	121	56
Water	0	0	0	0	0

Note. FB: Flavored Beverage.

**Table 2:** Participants Characteristics.

	Boys	Girls	Total
Sample Size, *n*	10	11	21
Age, y	9.4 ± 0.8	8.8 ± 0.9	9.1 ± 0.9
Height, m	1.37 ± 0.01	1.33 ± 0.07	1.35 ± 0.09
Weight, kg	30.3 ± 5.5	29.1 ± 5.5	29.6 ± 5.4
BMI, kg/m^2^	15.9 ± 1.1	16.3 ± 2.2	16.1 ± 1.7
Body fat, %	23.9 ± 5.4	29.4 ± 11.4	28.8 ± 10.3
Fat-free mass, kg	22.4 ± 4.7	21.7 ± 6.6	22.6 ± 6.3
Total body water, L	16.8 ± 3.4	15.9 ± 4.0	16.3 ± 3.6
Extracellular fluid, L	8.4 ± 1.2	7.4 ± 1.8	7.9 ± 1.6
Intracellular Fluid, L	8.4 ± 2.5	8.6 ± 2.4	8.5 ± 2.5

Note. Values represent mean ± standard deviation. BMI: body mass index.

**Table 3:** Urine Variables.

Time, Min	0	60	120	180
Urine Specific Gravity				
FB	1.010 ± 0.007	1.004 ± 0.004	1.003 ± 0.003 *	1.004 ± 0.007 *
Water	1.010 ± 0.007	1.008 ± 0.007	1.010 ± 0.008	1.009 ± 0.009
Urine Color				
FB	3 ± 2	2 ± 1	1 ± 1 *	2 ± 1 *
Water	3 ± 2	2 ± 2	3 ± 2	3 ± 2

Note. Values represent mean ± SD. FB: Flavored Beverage. *, denotes a statistically significant difference compared to Water for the same time point (*p* < 0.05).

**Table 4:** Perceptual Drink Scores.

Time, Min	0	25	60	85	120	145	180
Thirst, mm							
FB	36 ± 32	50 ± 51	44 ± 35	49 ± 37	59 ± 47	31 ± 38	36 ± 37
W	48 ± 52	59 ± 41	46 ± 36	59 ± 41	46.0 ± 36	60 ± 52	37 ± 39
Stomach Fullness, mm							
FB	60 ± 37	60 ± 44	42 ± 32	27 ± 27	28 ± 29	21 ± 29	16 ± 20
Water	54 ± 37	53 ± 40	47 ± 35	25 ± 27	18 ± 21	12 ± 23	14 ± 23
Mouth Dryness, mm							
FB	36 ± 36	46 ± 49	32 ± 33	29 ± 31	40 ± 39	37 ± 42	32 ± 37
Water	34 ± 34	51 ± 50	37 ± 36	50 ± 52	24 ± 41	51 ± 48	44 ± 36

Note. Values represent mean ± SD. FB: Flavored Beverage.

## Data Availability

The data presented in this study are not publicly available due to privacy and ethical restrictions related to participant confidentiality.

## Supplementary Materials

The following supporting information can be downloaded at: https://www.mdpi.com/article/10.3390/nu17152418/s1, Table S1: Descriptive statistics (Mean ± SD) for all outcome variables across time points in both conditions. FB: Flavored Beverage

## Abbreviations

The following abbreviations are used in this manuscript:

NHANES	National Health and Nutrition Examination Survey
USG	urine specific gravity
SSBs	sugar-sweetened beverages
NNSs	non-nutritive sweeteners
FDA	US Food and Drug Administration
