# Supplementary material for: A Low-Sugar Flavored Beverage Improves Fluid Intake in Children During Exercise in the Heat
Source: Nutrients. 2025 Jul 24;17(15):2418. doi: 10.3390/nu17152418 (PMC12348641; doi:10.3390/nu17152418)
Supplement: Supplementary file 1 [file nutrients-17-02418-s001.zip › nutrients-3758119-supplementary.pdf]

Table S1. Descriptive statistics (Mean±SD) for all outcome variables across time points in both conditions. FB: Flavored Beverage.

Time, min	0	10	15	25	60	70	75	85	120	130	135	145	180
**Cumulative Fluid Intake, mL**													
**FB**	-	-	-	172±139	328±219	-	-	509±318	637±374	-	-	816±491	946±536
**Water**	-	-	-	75±85	188±152	-	-	318±207	380±201	-	-	461±231	531±268
**Net Fluid Balance, %**													
**FB**	0.0	0.3±0.7	0.4±0.8	0.5±0.9	0.3±1.0	0.4±1.2	0.4±1.3	0.4±1.4	0.0±1.3	0.1±1.3	0.2±1.3	0.3±1.4	-0.6±1.3
**Water**	0.0	0.0±0.1	0.0±0.1	0.1±0.2	-0.4±0.7	-0.2±0.7	-0.1±0.8	-0.2±-0.8	-0.5±0.8	-0.5±0.8	-0.4±0.8	-0.5±0.9	-0.9±0.7
**Urine Volume, mL**													
**FB**	86±68	-	-	-	188±111	-	-	-	266±131	-	-	-	273±168
**Water**	133±90	-	-	-	148±147	-	-	-	120±111	-	-	-	133±134
**Cumulative Urine Volume, mL**													
**FB**	86±68	-	-	-	188±111	-	-	-	454±180	-	-	-	727±292
**Water**	133±90	-	-	-	148±147	-	-	-	268±212	-	-	-	401±293
**Urine Osmolality, mmol/kg**													
**FB**	524±277	-	-	-	196±154	-	-	-	130±108	-	-	-	177±220
**Water**	441±301	-	-	-	345±272	-	-	-	420±313	-	-	-	369±300
**Urine Osmotic Excretion, mmol**													
**FB**	36±23	-	-	-	24±7	-	-	-	24±10	-	-	-	24±10
**Water**	47±50	-	-	-	30±13	-	-	-	37±66	-	-	-	37±66
**Cumulative Osmotic Excretion, mmol**													
**FB**	36±23	-	-	-	24±7	-	-	-	48±15	-	-	-	72±23
**Water**	47±50	-	-	-	30±13	-	-	-	66±39	-	-	-	99±85
**Likeness, mm**													
**FB**	123±42	-	-	120±71	126±37	-	-	121±41	114±41	-	-	114±48	112±56
**Water**	62±46	-	-	71±48	74±51	-	-	69±56	73±52	-	-	82±62	82±48
